# Primary non leukemic myeloid sarcoma of the ureteral wall: a case report of a rare disease

**DOI:** 10.1093/jscr/rjad433

**Published:** 2023-08-22

**Authors:** Luigi Quaresima, Giordano Polisini, Daniela Fasanella, Vanessa Cammarata, Andrea Benedetto Galosi, Willy Giannubilo

**Affiliations:** Department of Urology at the Civitanova Marche Hospital, Civitanova Marche, Italy; Department of Clinical and Specialist Sciences, Division of Urology, Polytechnic University of the Marche Region Medical School, Ancona, Italy; Department of Life, Health and Environmental Sciences, University of L'Aquila, L'Aquila, Italy; Department of Clinical and Specialist Sciences, Division of Urology, Polytechnic University of the Marche Region Medical School, Ancona, Italy; Department of Clinical and Specialist Sciences, Division of Urology, Polytechnic University of the Marche Region Medical School, Ancona, Italy; Department of Urology at the Civitanova Marche Hospital, Civitanova Marche, Italy

**Keywords:** myeloid sarcoma, ureter, ureteral neoplasm, ureterectomy, hydronephrosis

## Abstract

Myeloid sarcoma (MS) is an extramedullary tumor mass causing proliferation of mature or immature blast cells of one or more myeloid lineages. Involvement of the genitourinary tract is rare. We present a case of MS of the ureteral wall. A 74-year-old man was evaluated for left hydronephrosis and ipsilateral low back pain. A computed tomography scan showed a nodular formation in the pelvic ureter. Urinary cytology revealed cellular atypia, so ureteroscopy was performed showing a distal ureteral mass. The histological examination of the biopsy revealed to be malignant neoplasm. The patient underwent left laparoscopic nephroureterectomy with bladder cuff excision. Microscopic histological examination revealed a tumor compatible with MS. A postoperative positron emission tomography revealed residual hypercaptation of the bladder, pelvic muscle and iliac nodes, so the patient started chemotherapy. A multidisciplinary approach was required, taking into account the patient’s age, the already poor renal function and the location of the neoplasm.

## INTRODUCTION

Myeloid sarcoma (MS) is defined as an extramedullary tumor mass that causes the proliferation of mature or immature blasts of one or more of the myeloid lines [1, 2].

MS typically occurs simultaneously with acute myeloid leukemia (AML), but it can also present in isolated forms or *de novo* as manifestation of relapsed AML, progression of a myelodysplastic syndrome, chronic myeloid leukemia and other myeloproliferative syndromes [1, 3].

MS is mildly predominant in males and the most commonly affected structures are the lymph nodes, skin, soft tissue, bones, gastrointestinal tract and central nervous system [1–7]. The genitourinary tract is rarely involved [5–7].We herein present a case of MS of the ureteral wall. To the best of our knowledge, only one case of primary ureteral MS has been reported so far [5].

## CASE PRESENTATION

**Figure 1:**
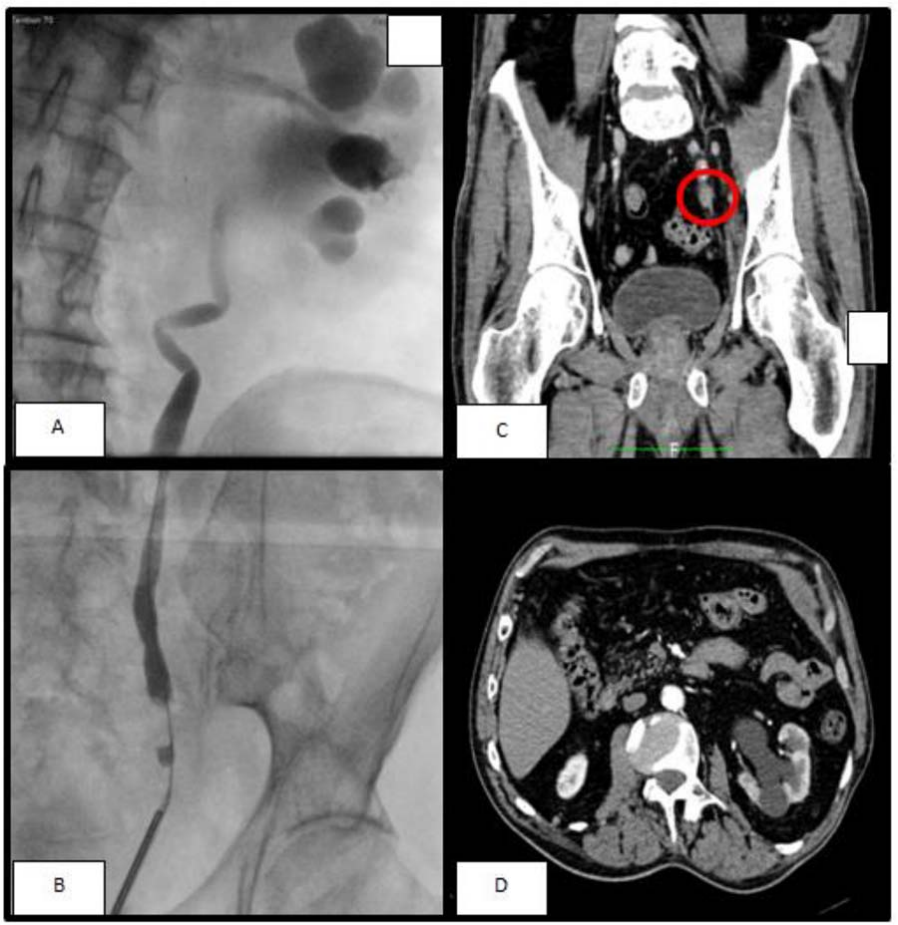
(**A, B**) The retrograde pyelography, performer before ureteroscopy, showed a minus contrast at the level of the pelvic ureter with an upstream hydroureteronephrosis; (**C**) left ureteral mass seen at arterious CT-scan; (**D**) the arterious CT-scan showed a hydronephrosis with corticalization of the calyxes.

A 74-year-old man was seen for left hydronephrosis and ipsilateral lumbar pain. A contrast-enhanced abdominal computed tomography (CT) scan showed left hydroureteronephrosis with a reduction of ipsilateral renal function caused by a solid nodular formation of about 33 mm in the pelvic ureter characterized by enhancement and hyperdensity of surrounding fat (Fig. 1). So, the patient performed a positron emission tomography (PET) scan with 18-Fluorodeoxyglucose (FDG) that showed uptake of the radiopharmaceutical Standardized Uptake Value (SUV) 4.38–5.32 in the area described on the abdomen CT. Urinary cytology revealed atypical urothelial cells. Therefore, we performed a ureteroscopy with biopsy that showed a solid distal ureteral mass involving almost the entire ureteral lumen. Histological examination of biopsy revealed a malignant neoplasm.

Since the outcomes of histological examination, the reduction of renal function and the advanced age, we decided to perform left laparoscopic nephroureterectomy with bladder cuff excision. The defined histological examination revealed a malignant neoplasm. The tumor was compatible with MS of the distal part of the ureter. Immunohistochemically neoplastic cells were CD117 + myeloperoxidase + CD99 + FLI1 + CD34 +/− TdT −/+ GATA3 −/+ cytokeratins AE1/AE3 – cytkeratins 8/18 – CD4 – CD56 – PAX5 – CD2 – CD3 – CD5 – tryptase–.

The bone marrow biopsy did not show the presence of disease (cellularity 40%, CD34 + and CD117 + blasts <5%, cell plasma population <10%). In addition, a bone marrow aspirate and a flow cytometry were performed and they were resulted both normal.

The cytogenetic investigation of bone marrow revealed a normal 46XY karyotype. Quantitative molecular analysis of the WT-1 gene was negative and the search for mutations of NPM1, FLT3 / TKD and FLT3 / ITD genes were wild type. In conclusion, all these investigations excluded the presence of an AML.

The patient also repeated a postoperative PET that disclosed residual bladder, pelvic muscle and iliac node hypercaptation and he started chemotherapy with Decitabine (demethylating agent) and Venetoclax (inhibitors of BCL2) to treat the MS and to prevent a future AML.

Next follow-up schedules after the third cycle of chemotherapy showed absence of recurrence of the neoplasm.

## DISCUSSION

MS is a hematological solid neoplasm. It is mildly prevalent in males and affects several parts of the human body, including the genitourinary tract. In fact in the literature are described cases of MS of the kidney, prostate, epididymis, testes, bladder, cervix and ureter [4–13].

The most frequently expressed markers in MS are CD68-KP1, MPO, CD117, CD99, CD68 / PG-M1, lysozyme, CD34, terminal dexynucleotidyltransferase, CD56, CD61, CD 30, glycophorin A, CD4 and others [1, 3]. The most commonly chromosomal abnormalities associated with MS are t(8; 21), monosomy 7, trisomy 8, trisomy 11, trisomy 4, inversion (16), 5q deletion etc [1, 3]. The most commonly mutated genes in MS are NPM1 (the most frequent), FLT3-ITD, IDH2, NRAS, KRAS, KIT etc [1, 3].

In our case the endoscopic biopsy, performed before surgery, showed an undifferentiated tumor of not otherwise determinable. This was due to the consumption of the histologic substrate following an ‘algorhitmic’ immunohistochemical approach. Thus, if a small biopsy fragment from the ureter is submitted to histological examination, we suggest immediately preparing at least 10 tissue sections, with the first and the last stained with hematoxylin–eosin; the immunohistochemical panel must ideally encompass cytokeratins, synaptophysin, CD2, CD3, CD20, PAX5, CD34 and CD117; depending on the amount of the tissue substrate, Ki67, TTF1 and GATA3 are also desirable.

Due to the advanced age of the patient and mostly to the reduced function of the kidney resulted from a long-term obstruction, we preferred to perform a left nephroureterectomy with bladder cuff excision.

In the only paper about primary ureteral MS, authors opted for a distal ureterectomy and ureteral reimplantation because of the young age of the patient and the possibility of benign neoplasm (the pathological examination was not informative, in their case due to artifacts).

**Figure 2:**
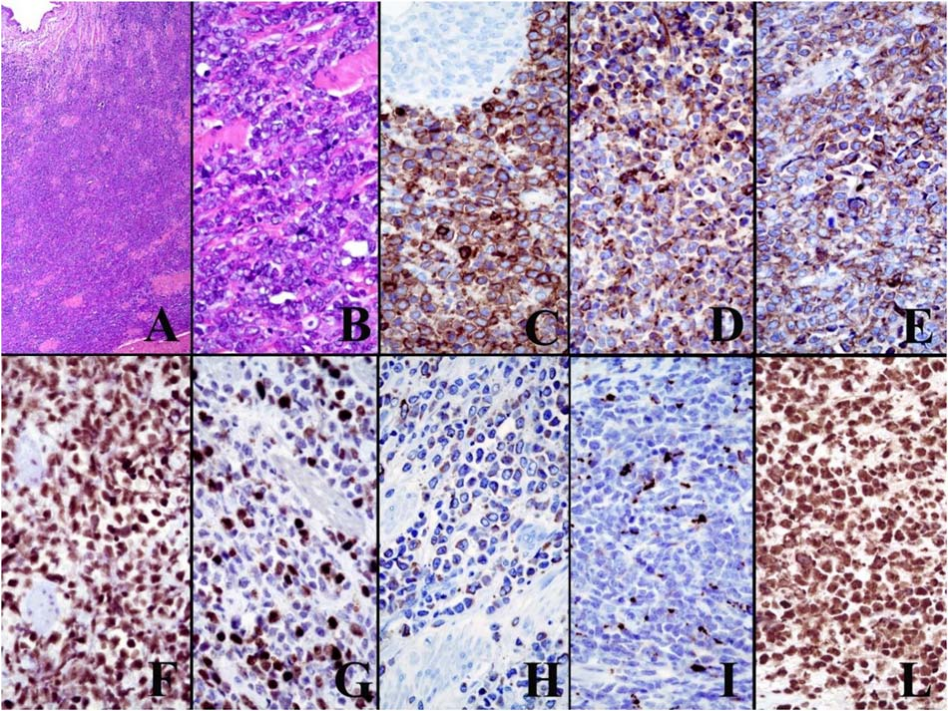
The immunohistochemical panel. The final histological examination showed microscopically a MS with the following immunohistochemistry: CD117 +, myeloperoxidase +, CD99 +, FLI1 +, CD34 +/−, TdT +/−.

The final histological examination showed macroscopically a dilated calico-pielic system with a widely padded ureteral wall and microscopically a MS with the following immunohistochemistry: CD117 +, myeloperoxidase +, CD99 +, FLI1 +, CD34 +/−, TdT +/− (Fig. 2). The subsequent bone marrow biopsy, aspirate and analyzes for chromosomal and genetic mutations were found negative. Therefore these data suggested that we were dealing with an isolated ureteral MS without concomitant AML or other myeloproliferative or myelodysplastic syndromes.

Regarding treatment, since virtually all patients with MS can develop AML, therapy for MS is based on the same protocols as that for AML, using chemotherapy, radiotherapy, surgery, hematopoietic stem cell transplantation and target therapies [1].

The cornerstone of treatment remains chemotherapy, which should be performed in all cases because it has been shown to reduce progression to AML even in isolated MS^2^ .

Surgery is useful to perform a biopsy to make a diagnosis and when the patient is symptomatic.

Radiation therapy is useful for local control of the disease and is generally used for MS located in the central nervous system or on the skin [2].

Bone marrow transplantation can be performed as a treatment for MS after induction of remission [2, 3].

Finally, there are new target therapies such as FLT3 inhibitors, DNA methyltransferaseinhibitors and anti-CD33 monoclonal antibodies, but, to date, the main treatment remains the chemotherapy [2, 3].

In conclusion, a multidisciplinary approach is warranted in order to achieve the best managing strategy for such a highly unusual malignancy.

## Data Availability

Being a case report, all data reported in the manuscript are accessible in the related Hospital of Civitanova Marche.
